# Impact on Autophagy and Ultraviolet B Induced Responses of Treatment with the MTOR Inhibitors Rapamycin, Everolimus, Torin 1, and pp242 in Human Keratinocytes

**DOI:** 10.1155/2017/5930639

**Published:** 2017-03-16

**Authors:** Song Xu, Li Li, Min Li, Mengli Zhang, Mei Ju, Xu Chen, Heng Gu

**Affiliations:** Institute of Dermatology, Jiangsu Key Laboratory of Molecular Biology for Skin Diseases and STIs, Chinese Academy of Medical Science & Peking Union Medical College, Nanjing, China

## Abstract

The mechanistic target of Rapamycin (MTOR) protein is a crucial signaling regulator in mammalian cells that is extensively involved in cellular biology. The function of MTOR signaling in keratinocytes remains unclear. In this study, we detected the MTOR signaling and autophagy response in the human keratinocyte cell line HaCaT and human epidermal keratinocytes treated with MTOR inhibitors. Moreover, we detected the impact of MTOR inhibitors on keratinocytes exposed to the common carcinogenic stressors ultraviolet B (UVB) and UVA radiation. As a result, keratinocytes were sensitive to the MTOR inhibitors Rapamycin, everolimus, Torin 1, and pp242, but the regulation of MTOR downstream signaling was distinct. Next, autophagy induction only was observed in HaCaT cells treated with Rapamycin. Furthermore, we found that MTOR signaling was insensitive to UVB but sensitive to UVA radiation. UVB treatment also had no impact on the inhibition of MTOR signaling by MTOR inhibitors. Finally, MTOR inhibition by Rapamycin, everolimus, or pp242 did not affect the series of biological events in keratinocytes exposed to UVB, including the downregulation of BiP and PERK, activation of Histone H2A and JNK, and cleavage of caspase-3 and PARP. Our study demonstrated that MTOR inhibition in keratinocytes cannot always induce autophagy, and the MTOR pathway does not play a central role in the UVB triggered cellular response.

## 1. Introduction

The mechanistic target of Rapamycin (MTOR) protein is a crucial signaling regulator in mammalian cells. Two types of MTOR containing complexes have been found in mammalian cells, MTOR complex 1 (MTORC1) and complex 2 (MTORC2), which are differently sensitive to Rapamycin and show different upstream and downstream signaling [1]. Currently, MTORC1 signaling has been discovered to be extensively involved in cellular biology, including autophagy [2], macromolecule biosynthesis [3], the cell cycle [4], growth [5], and metabolism [6].

Noticeably, the deregulation of MTOR signaling has been discovered to occur in human diseases, including cancer, diabetes, obesity, and neurodegeneration. Thus, there are many ongoing efforts to pharmacologically target this pathway [5]. Furthermore, the inhibition of the MTOR pathway has lengthened the lifespan in model organisms and has provided protection against many types of age related pathologies [7]. Importantly, the classical MTOR inhibitor Rapamycin has been found to prohibit the development of cutaneous squamous cell carcinoma in a transplanted patient population with immunosuppression [8].

The crosstalk between MTOR signaling and other cellular processes has been identified due to the increasing interest in MTOR function. For example, MTORC1 affects upstream and downstream endoplasmic reticulum (ER) stress signaling, while the latter can also facilitate or antagonize the output of MTORC1 signaling [9]. In addition, emerging evidence has revealed that the inhibition of MTOR signaling mediated the induction of apoptosis under various conditions; for instance, thymosin alpha 1 executed this effect in breast cancer [10]. It was reported that the inhibition of MTOR by pharmaceutical treatment, such as PF-04691502 [11], NVP-BEZ235 [12], and AZD8055 [13], can promote apoptosis, although these inhibitors affected MTOR activity through the indirect regulation of MTOR instead of the mediation of PI3K signaling. Indeed, some direct MTOR inhibitors have also been reported to induce apoptosis, for example, pp242 [14], temsirolimus [15], and everolimus [16]. Furthermore, it was reported that inhibiting MTOR activity can attenuate DNA damage and apoptosis [17]. Among the multiple target signaling pathways of MTOR, the autophagy process plays a key role in maintaining cellular homeostasis. Meanwhile, autophagy might mediate the biological effects caused by regulating MTOR signaling.

Keratinocytes are the most important structural cell type in the mammalian epidermis, which constitutes the first body barrier against various stressors and invasion [18]. The role of MTOR signaling in keratinocytes has not been fully clarified, although it has been reported to be involved in keratinocyte biology and pathology. Ultraviolet B (UVB) exposure, a common stressor of skin [19], is involved in various skin disorders such as sunburn [20], photocarcinogenesis [21], photoaging [22], and melanogenesis [23]. Importantly, UVB radiation was reported to increase the cascaded phosphorylation of MTOR substrate 4E-BP1 and its detachment from eIF-4E via the p38 pathway in the mouse epidermal cell line [24]. Additionally, UVB enhanced the phosphorylation of another MTOR substrate p70 S6 kinase, and this effect was inhibited by pretreatment with an MTOR inhibitor (Rapamycin), a PI3K inhibitor (LY294002), and an MEK/Erk inhibitor (PD98059) [25]. Furthermore, Rapamycin treatment prevented the increase in p70 S6 kinase phosphorylation at the early period after UVB stimulation in the human keratinocyte cell line HaCaT and dramatically decreased UVB-induced epidermal proliferation and cell cycle progression in a mouse model [26]. The UVB caused skin damage is involved in many types of cellular events such as DNA damage [27], apoptosis [28], ER stress [29], and activation of key signaling pathways (e.g., MAPK family [30], AMPK [31]). Considering the linkage between MTOR and these cellular machineries, one interesting question needs to be clarified whether inhibiting the MTOR pathway could affect the cellular events triggered by UVB radiation.

To clarify the role of MTOR signaling in keratinocytes, the preliminary work was to confirm the cellular responses to MTOR signaling inhibition. Although many MTOR inhibitors have been synthesized and utilized, reports concerning the responses to MTOR inhibitors except Rapamycin in keratinocytes are quite rare. Therefore, we first identified whether four widely used MTOR inhibitors, Rapamycin, everolimus, Torin 1, and pp242, work in the HaCaT human keratinocyte cell line and primary human epidermal keratinocytes (HEKs). Second, we determined the autophagy flux in the two keratinocytes following treatment with these MTOR inhibitors. Finally, we detected whether MTOR inhibitor treatment affects the cellular responses in the two keratinocytes exposed to UVB. As a result, keratinocytes were sensitive to the MTOR inhibitors Rapamycin, everolimus, Torin 1, and pp242, but the regulation of MTOR downstream signaling was distinct. Next, autophagy induction only was observed in HaCaT cells treated with Rapamycin but not in HaCaT cells treated with other three MTOR inhibitors. In addition, MTOR inhibition had no impact on the series of biological events in keratinocytes exposed to UVB.

## 2. Materials and Methods

### 2.1. Cells

As previously described [32], HaCaT cells were cultured in DMEM (Dulbecco's Modified Eagle's Medium) with 10% fetal bovine serum (both from Gibco, Invitrogen Corp., Carlsbad, CA, USA). The human primary epidermal keratinocytes (as previously described [33, 34]) were cultured in Keratinocyte SFM Medium (Gibco, Invitrogen Corp., Carlsbad, CA, USA).

### 2.2. Reagents and Antibodies

In this study, drugs and reagents included Rapamycin, 10 *μ*g/mL E64d, 10 *μ*g/mL pepstatin, Acridine Orange (AO), and dimethylsulfoxide (DMSO) (all from Sigma-Aldrich, St. Louis, MO, USA), Torin 1 (Tocris, Bristol, UK), pp242 (Abcam, Cambridge, MA, USA), everolimus (Cell Signaling Technology, Danvers, MA, USA), and FK-506 (tacrolimus) and pimecrolimus (both from Santa Cruz, Dallas, TX, USA). The control cells (the cells without drugs treatment or UVB radiation were named as nontreatment (NT)) were treated with 0.1% DMSO, which was used as the solvent for Rapamycin, E64d, pepstatin, Torin 1, pp242, everolimus, FK-506 (tacrolimus), and pimecrolimus. The DMSO solvent in our study was not beyond 0.1% [35].

### 2.3. UVB or UVA Radiation

Light source with lamps of UVB (Philips UVB Broadband PL-S 9W/12, Roosendaal, Netherland), delivering UV light between 290 nm and 320 nm, and peaking at 310 nm, was used in this study. At a distance of 16 cm, the mean irradiance of UVB was 1.50 mW/cm^2^, and the cells were exposed for 1, 3, 5, 6.7, 13.3, 20, and 33.3 seconds to 1.5, 4.5, 7.5, 10, 20, 30, and 50 mJ/cm^2^ of the irradiation dose. UVA (320 to 400 nm) was delivered from a solar simulator using a short-arc xenon lamp (Shanghai SIGMA High-Tech Co., Ltd., Shanghai, China). Interference filters were equipped for keeping the UVA integrity between 320 and 400 nm. At a distance of 16 cm, the mean irradiance of UVA was 38 mW/cm^2^, and the cells were exposed for 4 minutes and 23 seconds, 10 minutes and 57 seconds, and 21 minutes and 55 seconds to 10, 25, and 50 J/cm^2^ irradiation doses. Next, the cells were incubated in fresh DMEM with or without MTOR inhibitors after UVB or UVA exposure until lysis.

### 2.4. Western Blotting

RIPA Lysis buffer (Beyotime Biotechnology, Haimen, Jiangsu, China) including Protease Inhibitor Cocktail and phosphatase inhibitor PhosSTOP (both from Roche Applied Science, Basel, Switzerland) was used to lyse cells. After protein extraction, the BCA assay was performed to determine the total protein level in the supernatant of the cell lysate in each sample. Proteins in isoconcentration and isovolume were loaded on 4–12% NuPAGE Bis-Tris gels (Invitrogen Corp., Carlsbad, CA, USA) or 4–15% Mini PROTEAN TG precast polyacrylamide gels (Bio-Rad Laboratories, Hercules, CA, USA) and then were transferred into PVDF membranes (Bio-Rad Laboratories). Sequentially, the membranes were blocked in 3–5% bovine serum albumin solution and then were incubated with primary antibodies and secondary antibodies. Finally, the Chemiluminescence Imaging Method with ImmunStar WesternC Chemiluminescence Kit (Bio-Rad Laboratories) was used to visualize the protein bands. The intensities of certain protein bands (such as LC3A/B) were quantified with Quantity One. GAPDH was used as a loading control.

### 2.5. Cell Proliferation Assay

The bromodeoxyUridine (BrdU) cell proliferation ELISA kit was used according to the manufacturer's instructions (Number 11647229001, Roche Applied Science). Briefly, HaCaT cells were seeded in a 96-well plate with 20,000 cells per well and were cultured in the presence or absence of MTOR inhibitors for 36 hours. Next, 10 *μ*M BrdU was added in the culture medium, and incubation was continued for 3 hours. The incorporation of BrdU was determined by ELISA. According to the instruction, the incorporation of BrdU was calculated through the following formula: absorbance at 370 nm − absorbance at 492 nm.

### 2.6. Cell Migration Assay

The Oris™ cell migration assay kit (collagen I coated) was used according to the manufacturer's protocol (CMACC5.101, Platypus Technologies LLC, Madison, WI, USA). HaCaT cells were seeded at a density of 5 × 10^4^ cells per well in an Oris 96-well migration assay plate with cell seeding stoppers. Cells were cultured for 24 hours. The cell stoppers were removed, and 100 *μ*L of fresh medium with or without MTOR inhibitors was replaced, followed by incubation for 12 or 24 hours. The cell migration to the zone isolated by stoppers was observed, and the micrographs were captured under inverted microscopy. Cells in the migration zone were replicated for five independent experiments. The areas without cell migration per well were measured. The cell migration parameter was calculated using the following formula: (100%  −  areas without cell migration/area isolated by stopper) × 100.

### 2.7. AO Staining Assay

In view of autophagosomes, vacuole structures belonging to the acidic vesicular organelles (AVO), labeling AVO by AO staining was used to monitor autophagy [36, 37]. AVOs stained with AO were recorded using laser scanning confocal microscopy (FV1000, Olympus Corporation, Japan). The nuclei and cytoplasm of AO stained cells were visualized in deep and slight green fluorescence, while the AVOs in these cells were clearly marked as red fluorescence (AO G: *λ*_ex_ = 488 nm, *λ*_em_ = 515 nm; AO R: *λ*_ex_ = 546 nm, *λ*_em_ = 620 nm). In each cell, a higher intensity of red fluorescence implied a higher autophagy level. Therefore, to some extent, the intensity of red fluorescence might be measured to reflect the proportion to the volume of AVOs. The autophagy level in different treatment samples was measured with the average red/green fluorescence ratio per cell. The intensities of red and green fluorescence per cell were measured using Quantity One software. The mean red/green fluorescence ratios of different treatment cells were determined for at least three individual experiments, and the significant intergroup differences were analyzed statistically [36–38].

### 2.8. LC3B-GFP Puncta Analysis

To visualize the autophagy process, the LC3B-GFP transgene was added and transfected into HaCaT cells for protein expression via the Premo Autophagy Sensor LC3B-GFP BacMam 2.0 system (P36235, Invitrogen Corp., Carlsbad, CA, USA) according to the manufacturer's instructions. In this study, prior to the visualization, all cells were incubated with LC3B-GFP for at least 24 hours to enhance the efficiency of transfection. The intensity of LC3B-GFP puncta fluorescence in transfected cells was monitored and imaged with a laser scanning confocal microscope (GFP scanning: *λ*_ex_ = 530 nm, *λ*_em_ = 500 nm), and the number of LC3B-GFP puncta in transfected cells was determined using ImageJ software (http://imagej.nih.gov/ij/).

### 2.9. Cytotoxicity Measurement

To determine the cytotoxicity by MTOR inhibitors, UVB, or their treatments together, the Cell Counting Kit-8 (CCK-8) (Beyotime Biotechnology, Haimen, Jiangsu, China) was used according to the manufacturer's instructions [39, 40]. The cells were harvested into 24-well plates, and then the cells were treated with MTOR inhibitors, UVB, or both for indicated time. Next, 50 *μ*L of CCK-8 reagent was added to 500 *μ*L of medium, and the cells were then incubated for 2 hours at 37°C. The absorbance was measured using a microplate spectrophotometer at 450 nm.

### 2.10. Annexin V-EGFP Apoptosis Detection

Apoptotic cells were identified by the Annexin V-EGFP Apoptosis Detection Kit (Beyotime Biotechnology) as described previously [34]. The percentage of apoptotic cells was determined from three independent experiments.

### 2.11. Statistical Analysis

Individual experiments were performed at least three times, and similar results were obtained for statistical analysis. The data were analyzed with univariate ANOVA. Differences with *P* < 0.05 were identified to be statistically significant.

## 3. Results

### 3.1. Keratinocytes Are Sensitive to Treatment with the MTOR Inhibitors Rapamycin, Everolimus, Torin 1, and pp242

**Figure 1:**
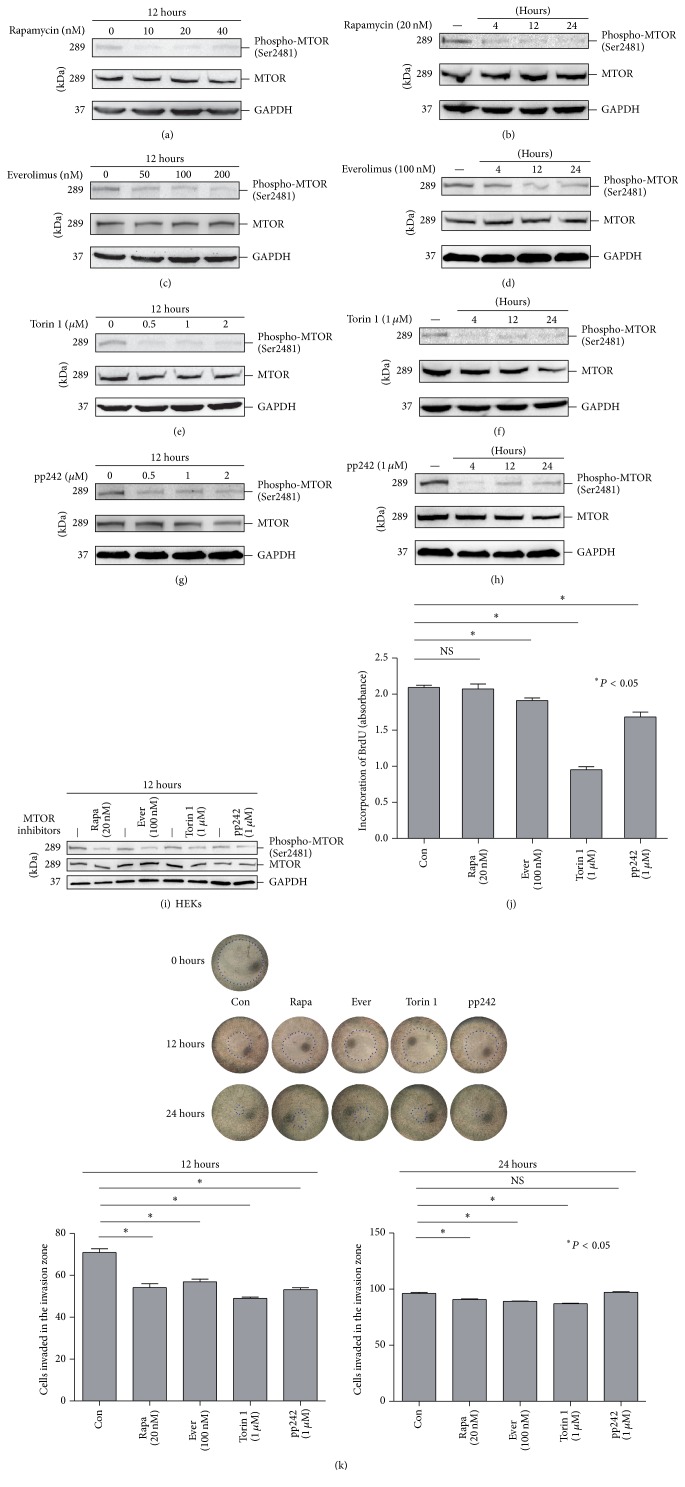
HaCaT cells were treated with or without different doses of Rapamycin ((a) 10, 20, and 40 nM), everolimus ((c) 50, 100, and 200 nM), Torin 1 ((e) 0.5, 1, and 2 *μ*M), or pp242 ((g) 0.5, 1, and 2 *μ*M) for 12 hours. Then, the HaCaT cells were treated with Rapamycin ((b) 20 nM), everolimus ((d) 100 nM), Torin 1 ((f) 1 *μ*M), or pp242 ((h) 1 *μ*M) for 4, 12, or 24 hours. Western blotting analysis was performed using primary antibodies against MTOR and phospho-Ser2481 mTOR. GAPDH served as a loading control. (i) HEKs were treated with or without Rapamycin (20 nM), everolimus (100 nM), Torin 1 (1 *μ*M), or pp242 (1 *μ*M) for 12 hours. HaCaT cells were treated with Rapamycin (20 nM), everolimus (100 nM), Torin 1 (1 *μ*M), or pp242 (1 *μ*M) for BrdU incorporation assay (j) and cell migration assay (k). The data were presented as means ± SD from three independent experiments and the representative figures were shown. Rapa: Rapamycin; Ever: everolimus; NS: nonsense.

To detect the sensitivity of HaCaT cells to MTOR inhibitor treatment, the cells were treated with different doses of Rapamycin (10, 20, and 40 nM), everolimus (50, 100, and 200 nM), Torin 1 (0.5, 1 and 2 *μ*M), or pp242 (0.5, 1, and 2 *μ*M) for 12 hours (Figures 1(a), 1(c), 1(e), and 1(g)). Next, the cells were treated with Rapamycin (20 nM), everolimus (100 nM), Torin 1 (1 *μ*M), or pp242 (1 *μ*M) for 4, 12, or 24 hours (Figures 1(b), 1(d), 1(f), and 1(h)). We found that the phosphorylation level of the MTOR protein, the core component of both MTORC1 and MTORC2, was decreased at the autophosphorylation site, Ser2481, which has been identified to monitor intrinsic MTOR specific catalytic activity [41]. The results were confirmed in HEKs (Figure 1(i)). These data suggested that HaCaT cells are sensitive to the treatment of these four MTOR inhibitors.

Moreover, BrdU incorporation and the cell migration assay were used to evaluate the effects on ribosomal biogenesis and growth of MTOR inhibitors in HaCaT cells. We found that, except for Rapamycin, everolimus, Torin 1, and pp242 exhibited different levels of inhibitory effect on DNA synthesis using the BrdU incorporation assay. Torin 1 showed the most significant effect (Figure 1(j)). Using the cell migration assay, we observed that treatment with the four MTOR inhibitors for 12 hours inhibited cell migration. However, the effects were rescued at 24 hours in cells treated with Rapamycin, everolimus, or Torin 1, and the inhibition on migration disappeared in pp242 treated cells (Figure 1(k)). These data suggested that inhibiting MTOR activity leads to the inhibition of proliferation and migration in HaCaT cells.

### 3.2. Effect of MTOR Inhibitors on Autophagy Flux

**Figure 2:**
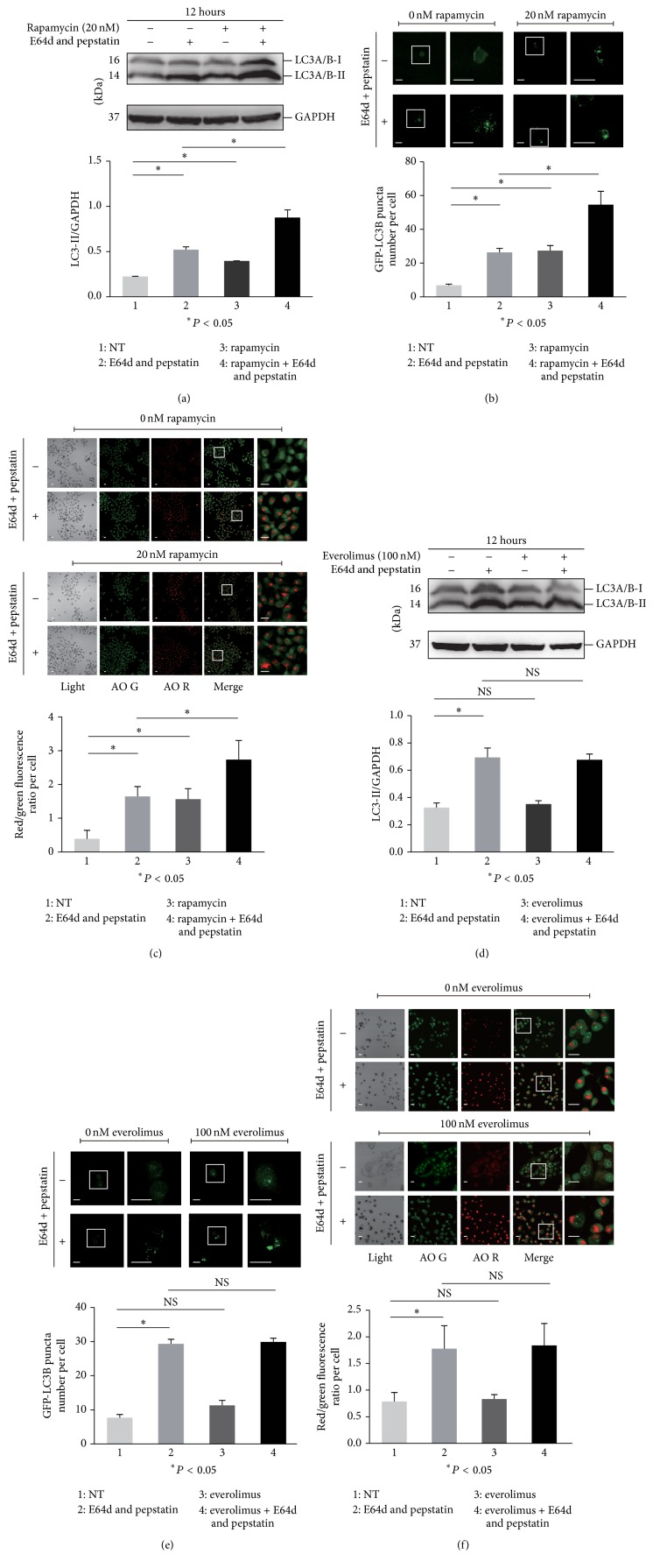
HaCaT cells were treated with or without 20 nM Rapamycin (a) or 100 nM everolimus (d) for 12 hours in the presence or absence of E64d (10 *μ*g/mL) and pepstatin (10 *μ*g/mL). Then, the cell lysate was subjected to determine the level of LC3 protein by western blotting. GAPDH served as a loading control. The ratios of LC3-II/GAPDH were calculated, and statistical differences between treatment and nontreatment (NT) were analyzed. HaCaT cells were pretreated with or without GFP-LC3B before Rapamycin (b) or everolimus (e) treatment for 12 hours in the presence or absence of E64d and pepstatin. HaCaT cells were treated with or without Rapamycin (c) or everolimus (f) for 12 hours in the presence or absence of E64d and pepstatin. Then, cells were incubated with AO. The cells (b, e, c, and f) were imaged by a laser scanning confocal microscope, and the means of GFP-LC3 puncta or red/green fluorescence ratios for individual cells were determined for statistical analysis. The data were shown as means ± SD from three independent experiments and the representative figures were shown. Bars = 20 *μ*m. NS: nonsense.

**Figure 3:**
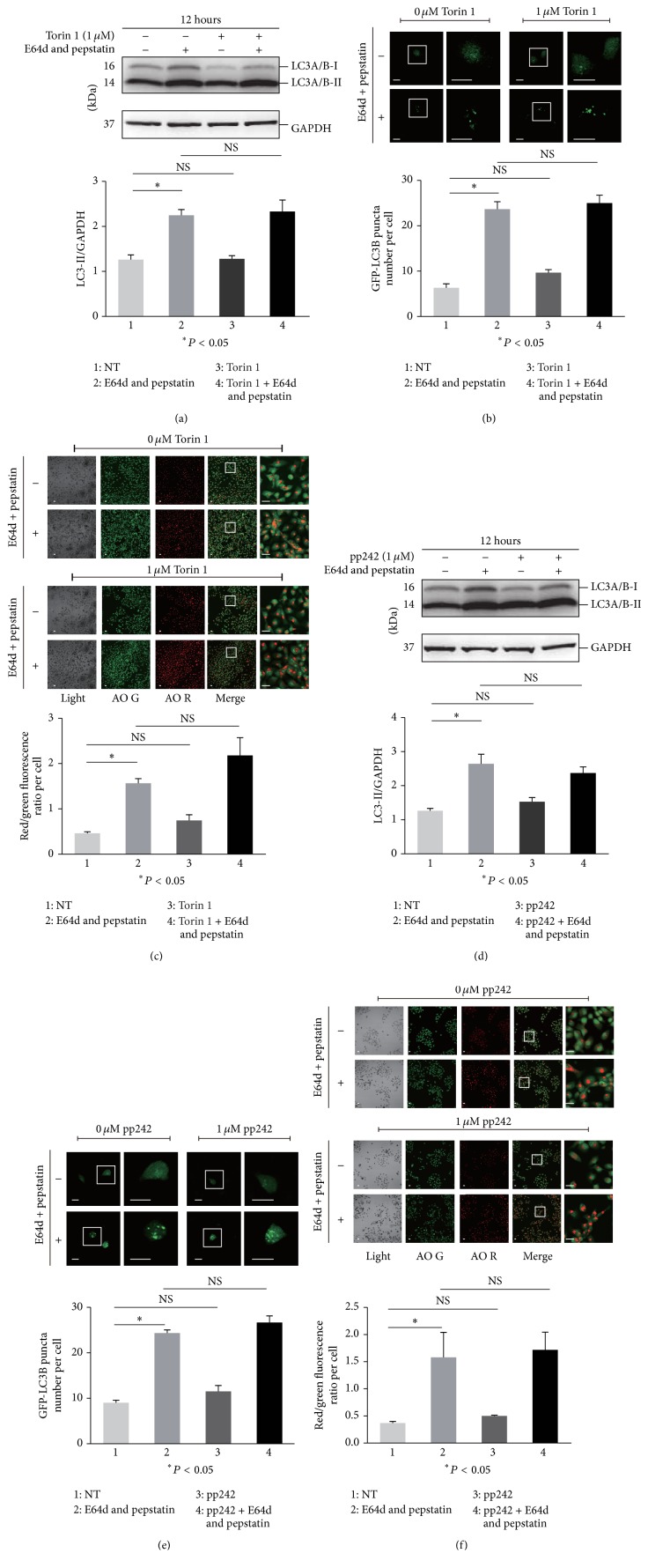
HaCaT cells were treated with or without 1 *μ*M Torin 1 (a) or 1 *μ*M pp242 (d) for 12 hours in the presence or absence of E64d and pepstatin. Then, the cell lysate was subjected to determine the level of LC3 protein by western blotting. GAPDH served as a loading control. The ratios of LC3-II/GAPDH were calculated, and statistical differences between treatment and nontreatment (NT) were analyzed. HaCaT cells were pretreated with or without GFP-LC3B before Torin 1 (b) or pp242 (e) treatment for 12 hours in the presence or absence of E64d and pepstatin. HaCaT cells were treated with or without Torin 1 (c) or pp242 (f) for 12 hours in the presence or absence of E64d and pepstatin. Then, cells were incubated with AO. The cells (b, e, c, and f) were imaged by a laser scanning confocal microscope, and the means of GFP-LC3 puncta or red/green fluorescence ratios for individual cells were determined for statistical analysis. The data were shown as means ± SD from three independent experiments and the representative figures were shown. Bars = 20 *μ*m. NS: nonsense.

The regulation of the autophagy process is one of the important biological functions of the MTOR pathway. To detect the autophagy flux, multiple methods were used in this study. First, the microtubule-associated protein 1 light chain 3 (LC3, a most widely used molecular marker of autophagy [42])-I to LC3-II conversion was determined in the presence or absence of the lysosome inhibitors E-64d and pepstatin, which were generally used in the autophagy flux assay due to their blockade of LC3-II degradation in autolysosomes [38]. We found an obvious increase in the ratio of LC3-II to the loading control GAPDH in Rapamycin treated HaCaT cells compared with that in untreated cells in the presence of E-64d and pepstatin, indicating the accumulation of newborn endogenous LC3-II (Figure 2(a)). Nonetheless, similar results were not observed in cells treated with Torin 1, pp242, and everolimus (Figures 2(d), 3(a), and 3(d)). The assay was replicated in HEKs, and all four MTOR inhibitors including Rapamycin did not increase the ratio of LC3-II/GAPDH in the presence of E64d and pepstatin compared with treatment alone with E64d and pepstatin after 12 hours of incubation, indicating the lower sensitivity of autophagy regulation to MTOR inhibitors in primary keratinocytes (Supplementary Figure 1(a) in Supplementary Material available online at https://doi.org/10.1155/2017/5930639). Next, GFP-LC3B puncta formation was detected to monitor autophagy in cells treated with MTOR inhibitors. We observed an increase in punctate GFP-LC3B in the presence of E-64d and pepstatin compared with its absence, suggesting the basal autophagy flux. The punctate GFP-LC3B was increased in Rapamycin treated cells compared with that in the control in the presence of E-64d and pepstatin, but not in cells treated with Torin 1, pp242, and everolimus (Figures 2(b), 2(e), 3(b), and 3(e)). Finally, the AO stained vacuoles were measured to analyze autophagosome formation [36, 37]. As a result, we found that the red/green fluorescence ratio per cell was increased in Rapamycin treated cells compared with that in control in the presence of E-64d and pepstatin. Similar results were not observed in cells treated with other MTOR inhibitors (Figures 2(c), 2(f), 3(c), and 3(f)). Collectively, these data demonstrated that only Rapamycin exhibited the effect of inducing autophagy among the four MTOR inhibitors tested in our study, although the crucial autophagy modulator, MTOR signaling, was inhibited in keratinocytes. The treatment doses of Rapamycin, everolimus, Torin 1, and pp242 were chosen according to those of previous studies [34, 43–45] and have been validated in the above work presented in Figure 1.

### 3.3. The MTOR Pathway in HaCaT Cells Is Sensitive to the MTOR Inhibitors Rapamycin, Everolimus, Torin 1, and pp242

**Figure 4:**
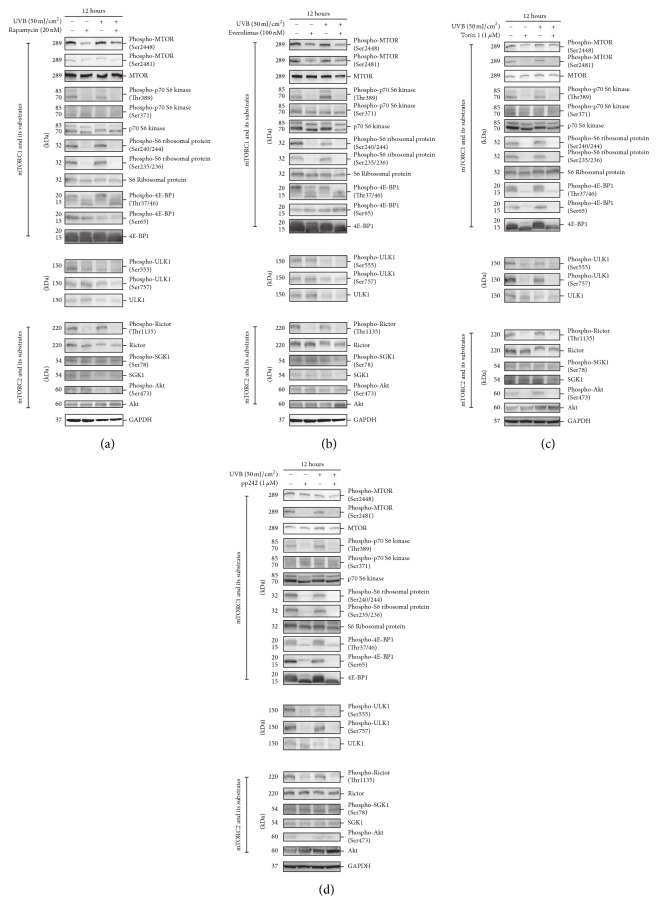
HaCaT cells were treated with or without 50 mJ/cm^2^ UVB and then incubated in the presence or absence of 20 nM Rapamycin (a), 100 nM everolimus (b), 1 *μ*M Torin 1 (c), or 1 *μ*M pp242 (d) for 12 hours. Western blotting was performed using primary antibodies against MTOR, phospho-Ser2448 or Ser2481 MTOR, p70 S6 kinase, phospho-Thr389 or Ser371 p70 S6 kinase, S6 ribosomal protein, phospho-Ser240/244 or Ser235/236 S6 ribosomal protein, 4E-BP1, phospho-Thr37/46 or Ser65 4E-BP1, ULK1, phospho-Ser555 or Ser757 ULK1, Rictor, phospho-Thr1135 Rictor, SGK1, phospho-Ser78 SGK1, Akt, and phospho-Ser473 Akt. GAPDH served as a loading control. Representative figures were exhibited from three independent experiments.

Although the decrease in MTOR phosphorylation is a molecular marker of these MTOR inhibitors, the targeted signaling and substrate of MTOR may be differentially affected due to the distinct pharmaceutic effect. Therefore, we detected the phosphorylation of downstream signaling mediators of MTOR besides the MTOR protein per se. In the downstream signaling of MTORC1, p70 S6 kinase and 4E-BP1 are direct substrates [46], and unc-51-like kinase 1 (ULK1) is phosphorylation modified by MTORC1 [47]. When MTOR signaling was activated or inactivated, these proteins presented the corresponding change in the protein levels or status of phosphorylation. We found that the phosphorylation levels of MTOR at Ser2448 and Ser2481 were decreased upon treatment with Rapamycin, Torin 1, pp242, and everolimus, suggesting that keratinocytes are sensitive to all four MTOR inhibitors (Figures 4(a)–4(d)). However, there were distinct alteration patterns of the downstream signaling of MTOR. On the one hand, the phosphorylation levels of 4E-BP1 and S6 Ribosomal protein, the substrate of p70 S6 kinase, were decreased in all cells treated with the four MTOR inhibitors. It is worth noting that Torin 1 and pp242 downregulated the 4E-BP1 expression on the protein level (Figures 4(c) and 4(d)). On the other hand, the decrease in ULK1 phosphorylation was observed in cells treated with Torin 1 and pp242, but not in cells treated with Rapamycin or everolimus. Interestingly, the core molecule of MTORC2, the Rapamycin-insensitive companion of mTOR protein (Rictor) [48], was dephosphorylated in cells treated with these MTOR inhibitors, but the phosphorylation level of its substrate serum and glucocorticoid induced protein kinase 1 (SGK1) [49] was not affected. Intriguingly, the phosphorylation of the MTORC2 target Akt was decreased in cells treated with Torin 1 or pp242 but not in cells treated with Rapamycin or everolimus.

These data indicated that different MTOR inhibitor treatments led to a distinct response of MTOR downstream signaling in keratinocytes such as ULK1 and 4E-BP1 signaling.

### 3.4. MTOR Inhibitors Did Not Affect UVB-Induced Cellular Responses, Including DNA Damage, the ER Response, and the JNK Signaling Pathway

To verify the role of MTOR signaling in UVB damage, we detected MTOR signaling in HaCaT cells exposed to UVB in the presence or absence of these MTOR inhibitors. Intriguingly, we did not observe the increase in MTOR phosphorylation, and the downstream targets of MTORC1 and MTORC2 including p70 S6 kinase, S6 ribosomal protein, 4E-BP1, ULK1, SGK1, and Akt in UVB challenged HaCaT cells at 12 hours after a 50 mJ/cm^2^ dose of exposure in the absence of MTOR inhibitors (Figures 4(a)–4(d)). The results were validated by the assay in HaCaT cells from 2 to 12 hours after 50 mJ/cm^2^ of UVB exposure (Supplementary Figure 1(b)). These data indicated that MTOR activity was not activated from 2 to 12 hours after 50 mJ/cm^2^ of UVB exposure. The UVB dose assay showed that a low level of UVB exposure (1.5, 4.5, and 7.5 mJ/cm^2^) activated MTOR activity, suggesting that the inactivation of MTOR activity in 50 mJ/cm^2^ UVB treated cells was not the artifact in experiment (Supplementary Figure 1(c)).

Furthermore, in the presence of these MTOR inhibitors, UVB treatment did not affect the inhibition of MTOR phosphorylation and its downstream signaling.

**Figure 5:**
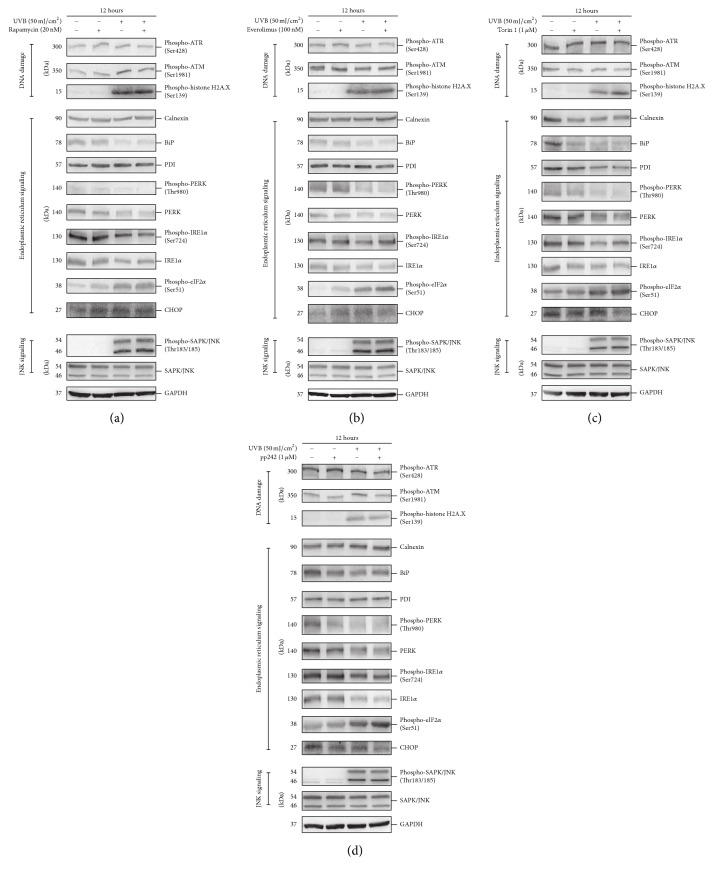
HaCaT cells were treated with or without 50 mJ/cm^2^ UVB and then incubated in the presence or absence of 20 nM Rapamycin (a), 100 nM everolimus (b), 1 *μ*M Torin 1 (c), or 1 *μ*M pp242 (d) for 12 hours. Western blotting analysis was performed using primary antibodies against phospho-Ser428 ATR, phospho-Ser1981 ATM, phospho-Ser139 H2A X, Calnexin, BiP, PDI, phospho-Thr980 PERK, PERK, phospho-Ser724 IRE1*α*, IRE1*α*, phospho-Ser51 eIF2*α*, CHOP, phospho-Thr183/185 SAPK/JNK, and SAPK/JNK. GAPDH served as a loading control. Representative figures were exhibited from three independent experiments.

Our previous study indicated that some UVB associated cellular events, such as apoptosis [34] and JNK activation (data not shown), were more significant in 50 mJ/cm^2^ UVB treated HaCaT cells. Furthermore, the apoptosis activation reached the peak at 12 hours after 50 mJ/cm^2^ of UVB exposure. Therefore, we treated cells with MTOR inhibitors for 12 hours to observe the effect on the cellular response by their treatment in UVB stimulated cells. DNA damage can activate a series of cellular signaling responses, including ataxia telangiectasia mutated kinase (ATM), ataxia telangiectasia and Rad3-related kinase (ATR), and Histone H2A family member H2A.X [50, 51]. We found that the phosphorylation of Histone H2A.X was significantly upregulated in UVB treated HaCaT cells, but the phosphorylation of ATR and ATM was not changed (Figures 5(a)–5(d)). These data suggested that the activation of Histone H2A.X is the more sensitive marker in UVB-induced DNA damage in keratinocytes.

Many types of molecular or physiological disturbances can impair ER function. Additionally, ER stress triggers the unfolded protein response (UPR), which is involved in regulatory signaling including protein kinase-like endoplasmic reticulum kinase (PERK) and inositol-requiring enzyme 1 *α* (IRE1*α*) [52]. The phosphorylation of both proteins was increased when ER stress occurred. We found that both the protein level and its phosphorylation of PERK and IRE1*α* were downregulated in UVB treated cells. The phosphorylation of eukaryotic initiation factor 2 *α* (eIF2*α*) is a well documented mechanism to decrease protein synthesis under stress conditions [53], and it can be phosphorylated at Ser51 by PERK [52]. We actually found that the phosphorylation of eIF2*α* was upregulated upon UVB treatment via a PERK independent mechanism. Therefore, our data indicated that UPR was inhibited, but protein synthesis was downregulated in HaCaT cells exposed to UVB radiation. Moreover, we also determined the level of some proteins which function as molecular chaperones to help protein fold properly, including calnexin [54], BiP [55], and protein disulfide isomerase (PDI) [56]. We observed that only the BiP was downregulated in UVB treated cells. The above data demonstrated that the normal function of ER was disturbed in HaCaT cells exposed to UVB damage (Figures 5(a)–5(d)).

Jun-amino-terminal kinase (JNK) (also named as stress-activated protein kinase, SAPK) is activated by various stimuli such as UV damage, inflammatory cytokines, and ceramides [57–59]. JNK pathway activation has been observed in UVB challenged keratinocytes [60, 61]. In accordance with previous reports, we observed JNK activation in UVB treated cells (Figures 5(a)–5(d)).

Importantly, the activation of Histone H2A.X, inhibition of PERK and IRE1*α* signaling, downregulation of BiP, and phosphorylation of eIF2*α* and JNK in UVB treated HaCaT cells were not restored by treatment with Rapamycin, everolimus, Torin 1, or pp242, suggesting that inhibiting MTOR signaling could not affect UVB-induced integrated cellular responses, such as DNA damage, ER function impair, and JNK activation. Interestingly, in the validated study using HEKs, we found that Rapamycin and everolimus did not affect the above UVB triggered cellular events in accordance with the observation in HaCaT cells. Torin 1 and pp242 cannot restore the inhibition of PERK and IRE1*α* signaling and downregulation of BiP, although Torin 1 interestingly inhibited the phosphorylation of Histone H2A.X and JNK activation (Supplementary Figure 2(a)). The observation in HEKs confirmed that inhibiting MTOR signaling might not be considered as a target to shield the cellular response to UVB radiation.

To investigate the effect on MTOR activity by ultraviolet light, we further detected the cellular events in the presence or absence of MTOR inhibitors in HaCaT cells treated with another important spectrum of solar ultraviolet UVA. First, we found that MTOR phosphorylation was decreased after 25 to 50 J/cm^2^ UVA exposure, suggesting the sensitivity of MTOR to UVA in contrast to the UVB radiation (Supplementary Figure 2(b)). Moreover, 50 J/cm^2^ UVA exposure led to the increase in Histone H2A.X phosphorylation and JNK activation but did not inhibit the expression of BiP, PERK, or IRE1*α* like UVB (Supplementary Figure 2(c)). Interestingly, four MTOR inhibitors exhibited significantly different effects on cellular responses caused by UVA. For example, everolimus alleviated the UVA induced phosphorylation of Histone H2A.X, but Rapamycin aggravated this effect (Supplementary Figure 2(c)). These findings demonstrated that UVB and UVA led to different cellular effects, especially the response of MTOR signaling.

### 3.5. MTOR Inhibitors Did Not Affect Apoptotic Molecular Markers Associated with UVB Stimulation

**Figure 6:**
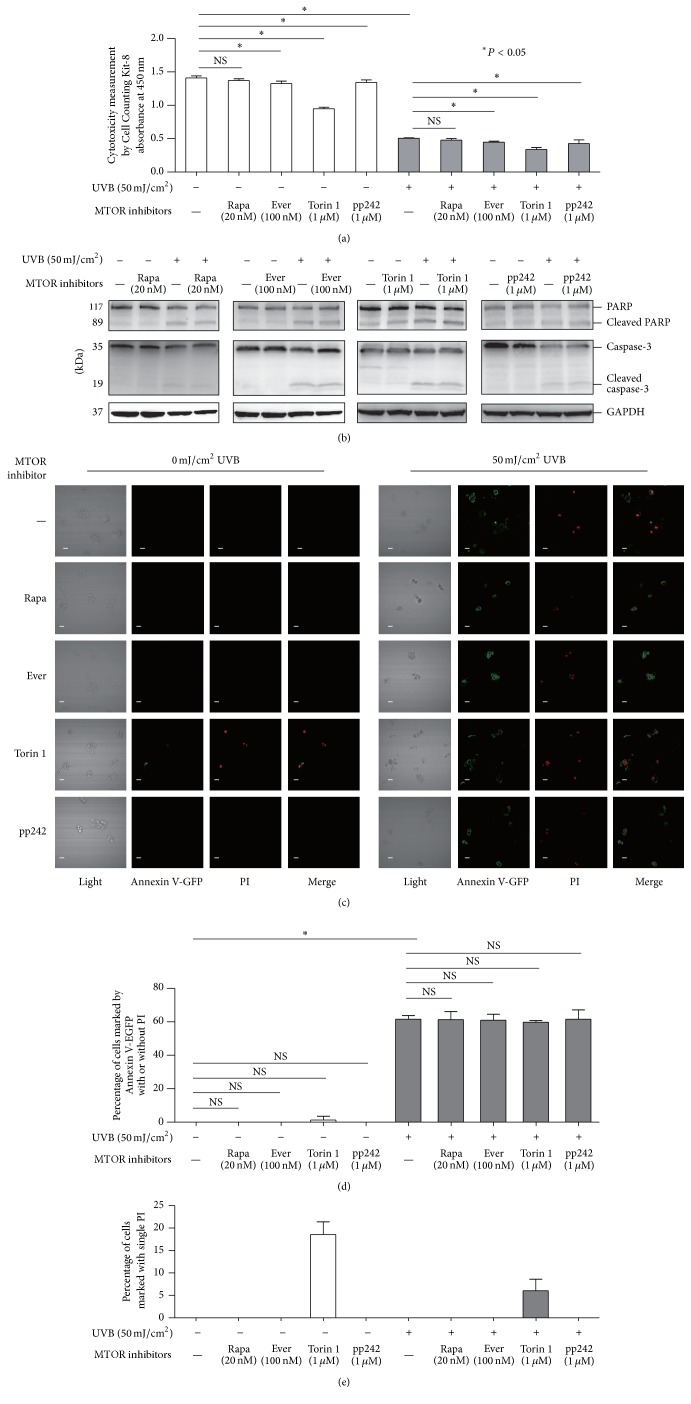
HaCaT cells were treated with or without 50 mJ/cm^2^ UVB and then incubated in the presence or absence of 20 nM Rapamycin, 100 nM everolimus, 1 *μ*M Torin 1, or 1 *μ*M pp242 for 12 hours. Cytotoxicity measurement was performed using Cell Counting Kit-8 (a). Western blotting analysis was performed using primary antibodies against PARP, cleaved PARP, caspase-3, and cleaved caspase-3 (b). GAPDH served as a loading control. The cells were imaged for Annexin V-EGFP apoptosis detection using a laser scanning confocal microscope (c). The percentages of cells marked by Annexin V-EGFP with or without PI (d) or single PI (e) were calculated. The individual experiment was performed three times, and the results were obtained for statistical analysis. Representative figures were shown from three independent experiments. Bars = 20 *μ*m. Rapa: Rapamycin; Ever: everolimus. NS: nonsense. ^*∗*^*P* < 0.05.

We found that MTOR inhibitor treatment (except Rapamycin) led to different levels of cytotoxicity on HaCaT cells. The impacts of everolimus and pp242 but not that of Torin 1 were slight. UVB radiation caused significant cytotoxicity in HaCaT cells, and MTOR inhibitor treatment after UVB exposure led to a more significant impact (Figure 6(a)). These findings demonstrated that inhibition of MTOR signaling did not rescue the cell damage caused by UVB. The above findings were validated in primary HEKs (Supplementary Figure 3(a)).

To further detect the impact of MTOR inhibitor treatment, we assessed the apoptotic markers caspase-3 and PARP in HaCaT cells with or without UVB challenge. Caspase-3 is a crucial executor of cellular apoptosis due to its critical role in proteolytic cleavage of various key proteins. The active form of caspase-3 contains two fragments, 17 kDa and 19 kDa, which are formed by its cleavage [62]. Poly(ADP-ribose) polymerase (PARP) is an important target of active caspase-3 during the apoptosis process [63], and cleaved PARP facilitates the disassembly of apoptotic cells [64]. Although MTOR inhibitors exhibited different levels of cytotoxicity, we found that each MTOR inhibitor treatment did not trigger apoptosis. In accordance with the results of previous studies [65, 66], we found the cleavage of caspase-3 and PARP in UVB treated HaCaT cells, suggesting UVB triggered apoptosis. Nonetheless, we found that UVB-induced activation of caspase-3 and PARP was not prohibited by any of the four MTOR inhibitors (Figure 6(b)). The above results were validated in HEKs (Supplementary Figure 3(b)). Furthermore, the ratios of cells stained with Annexin V alone and both Annexin V and propidium iodide (PI) were increased in UVB treated cells in the presence or absence of four MTOR inhibitors, and we did not observe a difference in cells stimulated by UVB in the presence or absence of the four MTOR inhibitors (Figures 6(c) and 6(d)). Our findings revealed that MTOR signaling was not involved in UVB triggered apoptosis. Interestingly, we observed an increase in staining with PI alone but not with Annexin V alone in Torin 1 treated HaCaT cells (Figures 6(c) and 6(e)), indicating that the 1 *μ*m Torin 1 treatment increased cell death was due to severe damage of the cell membrane. However, the Torin 1 induced cell death was not involved in apoptosis. Therefore, we did not find an increase in the cleavage of caspase-3 or PARP in Torin 1 treated cells.

In addition, transcription factor C/EBP homologous protein (CHOP) has been implicated in apoptosis in response to ER stress [67, 68]. We observed that CHOP was not affected upon UVB treatment (Figures 5(a)–5(d)), indicating that the ER stress mediated CHOP mechanism may not be related to UVB-induced apoptosis.

### 3.6. The Calcineurin Inhibitors Tacrolimus and Pimecrolimus Did Not Induce LC3-II Accumulation

**Figure 7:**
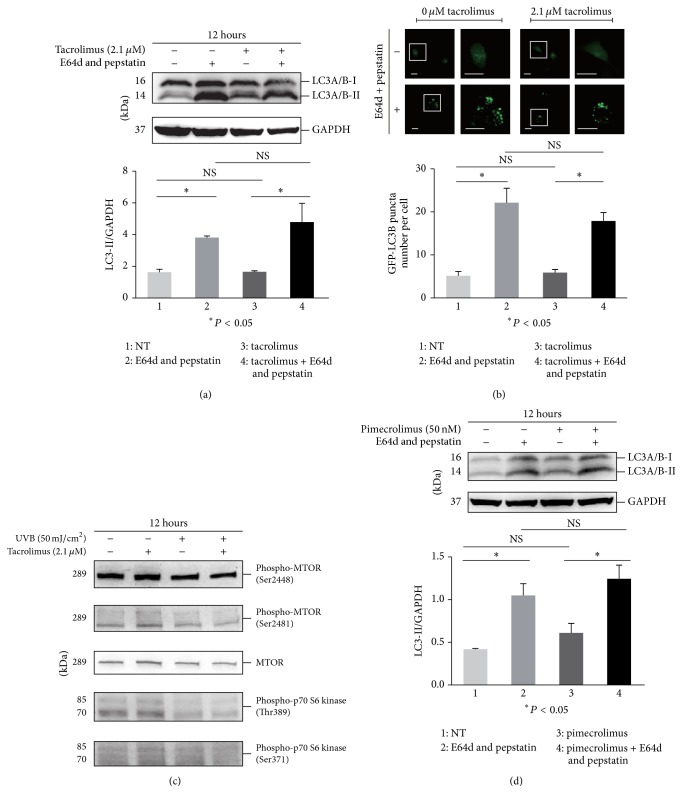
HaCaT cells were treated with or without 2.1 *μ*M tacrolimus (a–c) or 50 nM pimecrolimus (d) for 12 hours in the presence or absence of E64d and pepstatin. Then, the cell lysate was subjected to determine the level of LC3 protein (a and d) and MTOR and p70 S6 kinase as well as their phosphorylation (c) by western blotting. GAPDH served as a loading control. HaCaT cells were pretreated with or without GFP-LC3B before tacrolimus (b) treatment for 12 hours in the presence or absence of E64d and pepstatin. The cells (b) were imaged by a laser scanning confocal microscope, and the means of GFP-LC3 puncta for individual cells were determined for statistical analysis. The data were shown as means ± SD from three independent experiments and the representative figures were exhibited. Bars = 20 *μ*m. NS: nonsense.

Rapamycin is also known as another denomination sirolimus [69] and is used in combination with the calcineurin inhibitor tacrolimus as maintenance immunosuppressants in transplantation to selectively block the transcriptional activation of cytokines [43, 70, 71]. Importantly, Rapamycin binds two proteins, the FK506- (tacrolimus-) binding protein (FKBP) and FKBP-Rapamycin-associated protein (FRAP, the primal nomination of MTOR), in regulating cellular signaling [72, 73]. Thus, FKBP is the common target of both Rapamycin and tacrolimus. However, it is unclear whether tacrolimus can regulate autophagy flux and MTOR activity. We found that tacrolimus did not increase LC3-II accumulation, GFP-LC3 puncta, and the phosphorylation of MTOR and p70 S6 kinase (Figures 7(a)–7(c)). Our data indicated that tacrolimus may not affect autophagy and MTOR activity in keratinocytes, although it possesses the same cellular target as and similar pharmaceutic functions to Rapamycin. In addition, we found that pimecrolimus, which possesses a similar structure as tacrolimus, also did not increase LC3-II accumulation (Figure 7(d)). The treating doses of FK506 and pimecrolimus were chosen according to those in previous studies [74–76].

## 4. Discussion

Our study revealed that the MTOR signaling of human keratinocytes is sensitive to treatment with MTOR inhibitors, such as Rapamycin, everolimus, Torin 1, or pp242, but only the MTOR inhibition caused by Rapamycin can lead to autophagy induction. Moreover, the MTOR inhibition caused by Rapamycin, everolimus, or pp242 does not affect the series of biological events in UVB stimulated keratinocytes, including the downregulation of the ER molecular chaperone BiP and ER transmembrane protein PERK, activation of the DNA damage marker Histone H2A and stress-activated protein kinase SAPK/JNK, and cleavage of apoptotic molecular caspase-3 and PARP.

MTOR mediated regulation is the canonical autophagy machinery in mammalian cells, but it is unclear whether MTOR inhibition certainly results in autophagy induction. In this study, we first verified that both MTORC1 and MTORC2 signaling pathways are sensitive to these four MTOR inhibitors. Interestingly, Rictor of keratinocytes is sensitive to treatment with Rapamycin or everolimus, although it has been identified to be insensitive to Rapamycin [77]. Akcakanat et al. [78] found that Rapamycin treatment led to Rictor dephosphorylation in a time and concentration dependent manner, and their results were supported by our data. Complicated downstream pathways mediate MTOR signaling to modulate the autophagy process. It has been generally conceived that ULK1 (the homolog of autophagy-related gene 1 (ATG1) in yeast) protein plays a crucial role in the autophagy machinery downstream of MTOR signaling [79], but the role of ULK1 has not been clarified clearly. Reports regarding ULK1 in keratinocytes are rare. Recently, Akinduro et al. [80] reported that differentiating keratinocytes depleted of ULK1 lacked nucleophagy, and Kemp et al. [81] found that ULK1 signaling was deregulated by UV induced DNA damage. Our result is in accordance with the findings of Kemp et al., because we observed that ULK1 and its phosphorylation were inhibited after UVB stimulation. Our study preliminarily revealed the regulation of ULK1 in response to MTOR inhibitors in keratinocytes. First, ULK1 signaling is sensitive to Torin 1 and pp242 but insensitive to Rapamycin and everolimus, suggesting that ULK1 signaling is not unconditional in response to regulation by upstream MTOR. Second, ULK1 is not involved in Rapamycin induced autophagy, indicating that the ULK1 response is not indispensable for autophagy induction. Conclusively, the current findings demonstrate that a ULK1 independent mechanism exists in the autophagy machinery of keratinocytes. Our study reveals that canonical autophagy regulation has specificity in human keratinocytes. Importantly, our study indicated that Rapamycin is a more effective MTOR inhibitor as an inducer of autophagy in the treatment of human keratinocytes. Indeed, Qiang et al. [82] reported that Rapamycin induced autophagy and reduced UVB-induced tumorigenesis in mouse skin. These findings demonstrated the high availability of Rapamycin as an autophagy inducer for keratinocyte in vitro and in vivo.

Torin 1 and pp242 are potent blockers of MTOR activity through an ATP competitive mechanism [83]. Torin 1 [84] and pp242 [85] have been reported to induce autophagy on account of their inhibition of MTOR activity. However, we found that Torin 1 and pp242 treatment did not enhance autophagy flux in HaCaT cells, although the increase in the conversion from LC3-I to LC3-II was observed in cells treated with them. It is worth noting that Torin 1 has been reported to induce autophagy stronger than Rapamycin in mouse skin explants [80], indicating that we should continue to consider the availability of Torin 1 as an autophagy inducer* in vivo* study. Intriguingly, the similarities in MTOR signaling reaction existed between Torin 1 induced cascade and pp242 induced one and between Rapamycin induced cascade and everolimus induced one, but difference existed between two groups. These findings indicated that different MTOR inhibitor led to the different effects on the pathways related to MTOR and autophagy signaling. Therefore, it should be taken into consideration that nontarget effect like gene translation regulated by 4E-BP1 is different in utilization of MTOR inhibitors as the autophagy inducers. Indeed, some pathways were reported to be involved in MTOR independent autophagy regulation, for instance, inositol signaling [86], Ca2+/calpain, cAMP/Epac/Ins [87], JNK1/Beclin 1/PI3KC3 [88], and PKC [89]. Our previous study revealed that trehalose, sucrose, and raffinose enhanced autophagy in keratinocytes through an MTOR independent way [34]. Therefore, the importance of MTOR independent should be taken into consideration in autophagy regulation of keratinocytes. However, aforementioned MTOR independent signaling pathways related to the keratinocyte autophagy machinery remain unclear, and more investigations should be performed.

Ultraviolet (UV) radiation is the common stressor in skin disorders. UV can be divided into UVA, UVB, and UVC according to the spectrum. Among them, UVB is closely correlated with epidermal cell photodamage, leading to sunburn, photoaging, DNA damage, and photocarcinogenesis [90–92]. Keratinocytes are the major target of UVB-induced skin damage because they serve as the predominant component in the epidermal structure. Bridgeman et al. [93] found that UVB radiation activated MTOR signaling in mouse epidermal keratinocytes and in mouse skin. Syed et al. [94] reported that UVB can increase MTOR phosphorylation at 1 hour after radiation. Carr et al. [26] and Tu et al. [95] reported that MTOR signaling activation can be observed after 2 hours of UVB exposure. Intriguingly, our data suggested that MTOR signaling may be restored to the basal level at 12 hours after the early time activation by UVB exposure. However, at the same observation time point, we still found the UVB triggered events such as the downregulation of the ER molecular chaperone BiP and ER transmembrane protein PERK, activation of the DNA damage marker Histone H2A and stress-activated protein kinase SAPK/JNK, and cleavage of apoptotic molecular caspase-3 and PARP in the presence or absence of each of the four MTOR inhibitors. Our findings demonstrated that MTOR signaling may not serve as the trigger to drive the UVB-induced cellular response. Interestingly, MTOR inhibition was observed in UVA treated HaCaT cells at the early time after exposure. Considering that human skin is simultaneously exposed to UVA and UVB from natural solar radiation, the associated effect on the MTOR pathway by exposure of UVB combined with UVA should be concerned in future studies of photodamage.

Nevertheless, inhibition of MTOR signaling has been observed to have the anticarcinogenesis potentiality in the UVB treated mouse model; for example, Rapamycin or apigenin treatment reduced UVB-induced epidermal proliferation through inhibiting MTOR activation [26, 93], and AZD4547 and Curcumin C3 complex suppressed UVB-induced epidermal hyperplasia via suppressing FGFR/MTOR signaling [96]. Hence, more work is needed to clarify the role of MTOR signaling in the network of UV regulated pathways, especially in the studies in vivo.

In this study, we only observed the increase in the Histone H2A family member H2A.X phosphorylation, which was involved in demarcation for reorganizing mammalian chromatin [51], but did not find significant changes in other DNA damage markers, such as ATR or ATM. We speculate that ATR and ATM are not key signaling components in keratinocytes in response to UVB stimulation. Indeed, Vogel and Herzinger reported that ATR and ATM were not essential for the checkpoint response to UVB [97]. Additionally, Lei et al. found that UVB-induced degradation of p21, which plays an important role in the cell cycle and DNA repair, did not require ATR, ATM, or both [98]. The ER signaling response in UVB irradiated keratinocytes is unclear because reports are lacking. However, Mera et al. found that the IRE1*α* downstream protein XBP1 was upregulated in HaCaT cells exposed to 10 and 20 mJ/cm^2^ UVB and that PERK was not phosphorylated [29]. They also found that polyubiquitination was also increased. Park and Jang reported that GRP78, an ER stress marker, was increased in HaCaT cells exposed to 200 or 400 mJ/cm^2^ UVB but not to 50 or 100 mJ/cm^2^ [99]. Therefore, we speculate that the ER signaling response is regulated in a dose dependent manner. Although the correlation between MTOR signaling and ER stress has been verified, our study indicates that MTOR inhibitors treatment does not rescue the UVB-induced ER signaling damage, including PERK and IRE1*α* inhibition and BiP downregulation. These data demonstrate that the MTOR pathway may not be involved in the ER response to UVB radiation. Wu et al. reported that JNK activation was required for apoptotic induction, and (+)-Catechin prevented UVB triggered apoptosis in keratinocytes through inhibiting JNK phosphorylation [100]. Our data verified that JNK activation is a key cellular event in cell photodamage because it has been observed in cells challenged with either UVB or UVA. However, MTOR inhibitors (especially Rapamycin, everolimus, or pp242) do not affect JNK activation in UVB or UVA treated keratinocytes. Therefore, we speculated that MTOR signaling does not play a crucial role in the complex cellular responses in keratinocytes with ultraviolet damage.

Our study only revealed the effect of MTOR activity inhibition on UVB triggered events by pharmaceutic approaches. The role of the MTOR pathway in keratinocytes exposed to UVB damage needs to be further demonstrated through genetic approaches to modulate MTOR signaling. In summary, our study demonstrated that MTOR inhibition in keratinocytes cannot always induce autophagy, and the MTOR pathway may not play an essential role in the UVB triggered cellular response. In addition, the roles of MTOR and its associated signaling, such as ULK1 signaling in the keratinocyte autophagy machinery, need to be clarified because keratinocytes may have the specificity in canonical autophagy regulation.

## Supplementary Material

Supplementary Figure.1: (a) HEKs were treated with E64d (10 μg/mL) and pepstatin (10 μg/mL) alone or with Rapamycin (20 nM), everolimus (100 nM), Torin 1 (1 μM) or pp242 (1 μM) in the presence of E64d and pepstatin. GAPDH served as a loading control. The ratios of LC3-II/GAPDH were calculated, and statistical differences between treatment and non-treatment (NT) were analyzed. (b) HaCaT cells were exposed to 50 mJ/cm^2^ UVB, and the cells were lysed at 2, 4, 8 and 12th hour after exposure. (c) HaCaT cells were treated with 1.5, 4.5, 7.5, 10, 20, 30 and 50 mJ/cm^2^ UVB, and the cells were lysed at 8^th^ hour after exposure. The MTOR phosphorylation was detected by western blotting (b and c). Representative figures were shown from three independent experiments. Rapa: Rapamycin; Ever: everolimus. NS: nonsense.
Supplementary Fig.2: (a) HEKs were exposed to 50 mJ/cm^2^ UVB and incubated in the presence or absence of 20 nM Rapamycin, 100 nM everolimus, 1 μM Torin 1 or 1 μM pp242 for 12 hours. The cell lysate was subjected to western blotting for detecting MTOR, Histone H2A.X, PERK, IRE1α and SAPK/JNK and the phosphorylation levels as well as the expression of Bip. (b) HaCaT cells were treated with 10, 25 and 50 J/cm^2^ UVA, and cells were lysed 1 hour after exposure. The MTOR expression and phosphorylation was detected by western blotting. (c) HaCaT cells were exposed to 50 J/cm^2^ UVA and incubated in the presence or absence of 20 nM Rapamycin, 100 nM everolimus, 1 μM Torin 1 or 1 μM pp242 for 12 hours. The cell lysate was subjected to western blotting for detecting MTOR, Histone H2A.X, PERK, IRE1α and SAPK/JNK and the phosphorylation levels as well as the expression of Bip. GAPDH served as a loading control.
Supplementary Fig.3: HEKs were treated with or without 50 mJ/cm^2^ UVB and then incubated in the presence or absence of 20 nM Rapamycin, 100 nM everolimus, 1 μM Torin 1 or 1 μM pp242 for 12 hours. Cytotoxicity measurement was performed using Cell Counting Kit-8 (a). Western blotting analysis was performed using primary antibodies against PARP, cleaved PARP, caspase-3 and cleaved caspase-3 (b). GAPDH served as a loading control.

## Abbreviations

MTOR: Mechanistic target of Rapamycin
ER: Endoplasmic reticulum
HEKs: Human epidermal keratinocytes
NGF: Nerve growth factor
VEGF: Vascular endothelial growth factor
HPV: Human papillomavirus
ULK1: Unc-51-like kinase 1
Rictor: Rapamycin-insensitive companion of mTOR protein
LC3: Microtubule-associated protein 1 light chain 3
AO: Acridine orange
UPR: Unfolded protein response
PERK: Protein kinase-like endoplasmic reticulum kinase
IRE1*α*: Inositol-requiring enzyme 1 *α*
eIF2*α*: Eukaryotic initiation factor 2 *α*
JNK: Jun-amino-terminal kinase
PARP: Poly(ADP-ribose) polymerase
CHOP: Transcription factor C/EBP homologous protein
DMSO: Dimethylsulphoxide
PI: Propidium iodide.
