# Supplementary material for: Impact on Autophagy and Ultraviolet B Induced Responses of Treatment with the MTOR Inhibitors Rapamycin, Everolimus, Torin 1, and pp242 in Human Keratinocytes
Source: Oxid Med Cell Longev. 2017 Mar 16;2017:5930639. doi: 10.1155/2017/5930639 (PMC5376460; doi:10.1155/2017/5930639)

Supplementary Fig.1

(a) HEKs

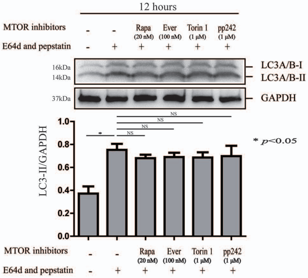

(b) HaCaT cells

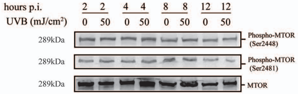

(c) HaCaT cells

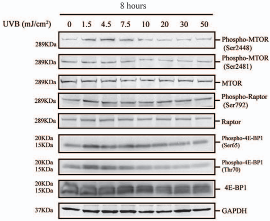

Supplementary Fig.2

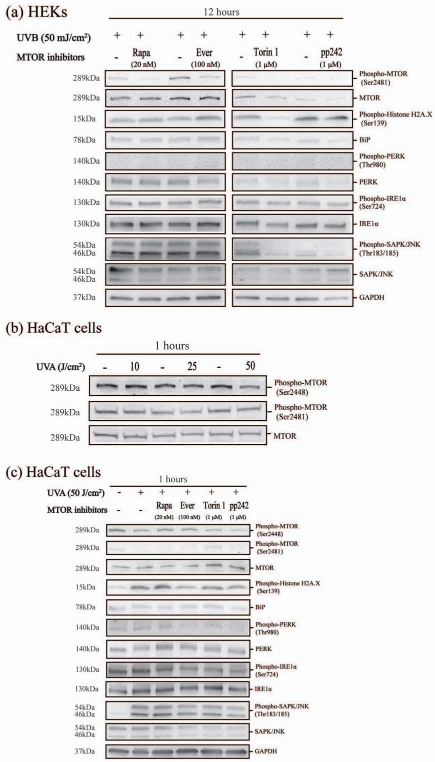

Supplementary Fig.3

(a) HEKs

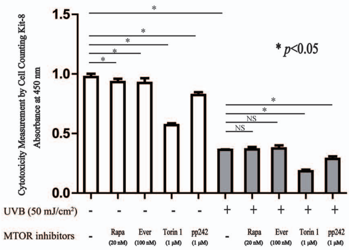

(b) HEKs

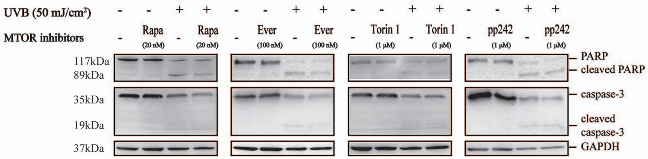

Supplement: Supplementary file 1 — Supplementary Figure.1: (a) HEKs were treated with E64d (10 μg/mL) and pepstatin (10 μg/mL) alone or with Rapamycin (20 nM), everolimus (100 nM), Torin 1 (1 μM) or pp242 (1 μM) in the presence of E64d and pepstatin. GAPDH served as a loading control. The ratios of LC3-II/GAPDH were calculated, and statistical differences between treatment and non-treatment (NT) were analyzed. (b) HaCaT cells were exposed to 50 mJ/cm2 UVB, and the cells were lysed at 2, 4, 8 and 12th hour after exposure. (c) HaCaT cells were treated with 1.5, 4.5, 7.5, 10, 20, 30 and 50 mJ/cm2 UVB, and the cells were lysed at 8th hour after exposure. The MTOR phosphorylation was detected by western blotting (b and c). Representative figures were shown from three independent experiments. Rapa: Rapamycin; Ever: everolimus. NS: nonsense. Supplementary Fig.2: (a) HEKs were exposed to 50 mJ/cm2 UVB and incubated in the presence or absence of 20 nM Rapamycin, 100 nM everolimus, 1 μM Torin 1 or 1 μM pp242 for 12 hours. The cell lysate was subjected to western blotting for detecting MTOR, Histone H2A.X, PERK, IRE1α and SAPK/JNK and the phosphorylation levels as well as the expression of Bip. (b) HaCaT cells were treated with 10, 25 and 50 J/cm2 UVA, and cells were lysed 1 hour after exposure. The MTOR expression and phosphorylation was detected by western blotting. (c) HaCaT cells were exposed to 50 J/cm2 UVA and incubated in the presence or absence of 20 nM Rapamycin, 100 nM everolimus, 1 μM Torin 1 or 1 μM pp242 for 12 hours. The cell lysate was subjected to western blotting for detecting MTOR, Histone H2A.X, PERK, IRE1α and SAPK/JNK and the phosphorylation levels as well as the expression of Bip. GAPDH served as a loading control. Supplementary Fig.3: HEKs were treated with or without 50 mJ/cm2 UVB and then incubated in the presence or absence of 20 nM Rapamycin, 100 nM everolimus, 1 μM Torin 1 or 1 μM pp242 for 12 hours. Cytotoxicity measurement was performed using Cell Counting Kit-8 (a). Western blotti [file 5930639.f1.pdf]
